# The New Nicotine Pouch Category: A Tobacco Harm Reduction Tool?

**DOI:** 10.1093/ntr/ntab198

**Published:** 2021-10-04

**Authors:** Sudhanshu Patwardhan, Karl Fagerström

**Affiliations:** 1 Centre for Health Research and Education, University of Southampton Science Park, Chilworth, UK; 2 Fagerstrom Consulting, Stockholm, Sweden

## Abstract

Nicotine pouches to be put under the upper lip are a new category of products that are being rapidly developed and marketed as consumer goods with little research or regulatory oversight. We have identified research gaps in assessing their harm and benefit potential, and possible regulatory science approaches to inform the policies that can allow a maximization of the category’s public health potential while minimizing unintended consequences.

Implications: This commentary presents a potential blueprint for a comprehensive assessment of the nicotine pouches category. Data from the proposed research areas can better inform the regulatory policy decisions around the category, with the aim to maximize the category’s tobacco harm reduction potential while minimizing unintended harms.

## Tobacco-Related Harms and Harm Reduction

Unburned tobacco contains about 16 carcinogens and tobacco smoke contains more than 60, most notably tobacco-specific nitrosamines, polycyclic aromatic hydrocarbons, and aromatic amines.^1^ There are over a billion users worldwide of higher risk forms of tobacco, consuming tobacco in smoked forms such as cigarettes, bidis, cigars, cigarillos and/or smokeless forms such as gutkha, zarda, and naswar.^2^ It is widely accepted that current available cessation products and services are suboptimal in their effectiveness. Cost and efficacy of current smoking cessation medications on the market is an impediment to availability, accessibility and cessation success in low-and-middle income countries (LMICs) where 80% of the world’s tobacco users live.^3^ This is compounded by the fact that in countries in South Asia, a predominant form of tobacco consumption is oral smokeless tobacco, especially among women and economically disadvantaged populations. Evidence-based cessation treatments and safer, affordable alternatives are not available for oral smokeless tobacco in these countries, widening the health inequity. Therefore, innovation in tobacco cessation products and services has the potential to reduce the societal impact of tobacco globally.^4^

## Oral Nicotine Pouch—Newer Nicotine Replacement Formulation

Recently new non-combustible products containing nicotine are rapidly entering the market. Examples of new product categories are electronic cigarettes, heat-not-burn products, and nicotine pouches (NP). This commentary discusses the oral NP category. The NP products are placed, like Swedish snus, between the upper lip and gum. The NP are different to Swedish-style snus in that there is no leaf tobacco in them. The precursor of today’s NP was studied in clinical studies as a new formulation for nicotine replacement (NR) treatment under the Zonnic brand in the late 2000s.^5^

The first NP to be widely distributed in the USA was branded ZYN that was marketed by Swedish Match North America. ZYN is a thin white pouch that contains white powdered nicotine. Other ingredients in ZYN include food-grade additives, fillers, a stabilizer (hydroxypropyl cellulose), pH adjusters, noncaloric sweeteners, and flavorings. Some other brands with mostly relatively similar compositions are Dryft, Loop, Lyft, Nordic Spirit, On!, Rouge, Rush, Velo, and ZoneX.

The NP have so far not been well researched and there are only a few papers published. One paper characterized ten different pouch products on variables relevant to uptake of nicotine such as pH, total nicotine content and protonated (free) nicotine. The authors suggest that users can draw adequate nicotine from the pouches to overcome cravings from cigarettes.^6^ The only published pharmacokinetics study on NP showed that despite a lower nicotine content, NP delivered nicotine as quickly and in a similar concentration compared to existing smokeless products. The authors also concluded that NP efficacy in reducing withdrawal symptoms and helping smokers reduce or stop combustible tobacco use should be similar or better than NR products.^7^ In another study, the toxicant levels of 26 harmful and potentially harmful constituents from three snus products, two NR products (gum and lozenge) and four Lyft (British American Tobacco) NP products were analyzed. Compared with snus, NPs had lower levels of 10 HPHCs and generally no difference could be seen between the two NR products and the four Lyft NP variants.^8^

In a consumer insight and user study of ZYN, it was found that the labeling and packaging of the product were such that almost 90% of never users and former users did not find it to be appealing. 3% of never users and 2% of former users were interested in buying the product. The majority of users were current smokeless tobacco (ST) users and former tobacco users. The most common reason for use of ZYN among current ST users was “less harmful to my health than other tobacco products.” ^9^

## Concerns with Nicotine Pouches

The situation in which the pouches exist today may change. From a product new to the market with limited reach and sales to a product more visible due to advertising, the attitude to the category may change among non-nicotine users. The marketing practices may become more aggressive and directed towards adolescents. To identify and address unintended consequences in a timely manner, further use studies and ongoing monitoring of these products are called for. Already, as per some investigative media reports, some of the manufacturers are practicing unethical market approach and targeting non-users and vulnerable young population for the sale of nicotine products.^10,11^ This brings into question the suitable regulatory regime for these products and the research gaps that need to be filled so as to inform a science-driven regulatory policy.

Oral NP come in an array of flavors and many believe that flavors can play a significant role in drawing youth to tobacco products and possibly be the primary reason to use the products.^12^

An escalating ‘nicotine strength’ war is another matter of concern. Around 2018, in Russia and some Eastern European countries, pouches were increasingly sold in much higher strengths (>20 mg/pouch) than needed for craving relief and withdrawal management in tobacco cessation. The NP category is now banned in Russia.^13^

## A Regulatory Science Agenda for Nicotine Pouches

Large gaps in NP related research still remain, before regulators, public health, and consumers can accept these as harm reduction products and another cessation tool. In order to be appropriate for the protection of public health the following research areas would give valuable insights for regulation and consumer information (Table 1).

**Table 1.** Regulatory Science Agenda for Nicotine Pouches

**Product chemistry** The product chemistry should be characterised with special emphasis on the flavourings’ potential for toxic effects.
**Pharmacokinetics** The pharmacokinetics should be characterised for different doses of nicotine and for different additives to the product that can alter the pH and the taste profile.
**What is the absolute and relative safety of the actual product itself?** For example, effect on the oral mucosa. If the product is marketed to users of combustible forms of tobacco, it would be important to compare the product’s safety with that of cigarettes.
**What is the acceptable maximum level of nicotine per pouch that can achieve its stated public health goal?** Since some of these products sold in certain countries had significantly higher doses of nicotine than seen with licensed medicinal NR products, there might be a need to have a limit on the nicotine dose taking into account the nicotine´s release and absorption from the product.
**Is the communication around the use of the product appropriately understood?** For example, that the pouches should be marketed and sold for use by current or former users of more risky tobacco products for complete substitution and not used to be together for long durations.
**Who are the intended consumers?** For example, that the pouches should be marketed solely to adult current users of more harmful products.
**What is the likelihood of unintended use in the population?** For example, if it is used by non-tobacco users and especially taken up by nicotine-naïve adolescents? If such uptake occurs it would be important to know if it is a gateway to other tobacco use.
**What is the transition time for current users to switch over to the product?** Understandably there may be a transition time among current users of more risky tobacco products from NP trial, to a phase of dual use (e.g. less than a year), to complete switch to pouches. That time needs to be understood, confirmed in large scale studies and communicated.
**Do these products deter or delay intentions to completely give up tobacco and nicotine use?** It will be important to study whether the marketing, sale and use of these products undermine individual level and population level attempts to quit risky forms of tobacco.
**Product abuse liability** Abuse liability should be studied preferably in comparison with other categories like cigarette smoking and ST.

## A Scientific Evidence Driven Regulatory Policy for Nicotine Pouches

Globally, manufacturers have a duty of care to address the prevalent research and knowledge gaps about this category. In a rapidly developing area such as innovative nicotine products, it is not surprising that regulators play catch up. Conducting NP related research and making the data available can better inform the regulatory policy.

Such an approach has the potential to accelerate the delivery of a wider range of affordable evidence-based cessation tools to LMICs such as India. India has the highest prevalence of oral cancers worldwide, driven largely by a vast array of risky ST products. With around 200 million current ST users, there is a great challenge and opportunity in India to bring affordable NP that are responsibly manufactured and marketed only to adult tobacco users. It would be important to ensure that adult users’ access and affordability are not compromised by the regulatory regime and the behaviors of profit-maximizing manufacturers.

In summary, NP is a new product category, close to the composition of ingredients to some NR products, that can be an effective tool for smokers, and other tobacco users, to reduce and stop tobacco use. A comprehensive regulatory science agenda will need to be prioritized and delivered by relevant stakeholders to maximize this category’s public health potential and minimize its unintended consequences.

## Supplementary Material

A Contributorship Form detailing each author’s specific involvement with this content, as well as any supplementary data, are available online at https://academic.oup.com/ntr.

ntab198_suppl_Supplementary_Taxonomy_Form
